# Neural Stimulation of Brain Organoids with Dynamic Patterns: A Sentiomics Approach Directed to Regenerative Neuromedicine

**DOI:** 10.3390/neurosci4010004

**Published:** 2023-01-16

**Authors:** Alfredo Pereira, José Wagner Garcia, Alysson Muotri

**Affiliations:** 1 Philosophy Graduate Program, UNESP/Marilia Campus, São Paulo State University, Botucatu 18618-689, Brazil; 2 Architecture and Urbanism, USP and PUC, São Paulo, Brazil; 3 Media Lab, MIT, Cambridge, MA, USA; 4 Stem Cell Program, Department of Pediatrics & Cellular Molecular Medicine/UCSD, Institute for Genomic Medicine, San Diego, CA, USA

**Keywords:** brain organoids, sentiomics, regenerative neuromedicine, dynamic patterns, Amazon Rainforest

## Abstract

The new science called *Sentiomics* aims to identify the dynamic patterns that endow living systems with the capacity to feel and become conscious. One of the most promising fields of investigation in *Sentiomics* is the development and ‘education’ of human brain organoids to become sentient and useful for the promotion of human health in the (also new) field of Regenerative Neuromedicine. Here, we discuss the type of informational-rich input necessary to make a brain organoid sentient in experimental settings. Combining this research with the ecological preoccupation of preserving ways of sentience in the Amazon Rainforest, we also envisage the development of a new generation of biosensors to capture dynamic patterns from the forest, and use them in the ‘education’ of brain organoids to afford them a ‘mental health’ quality that is likely to be important in future advances in ‘post-humanist’ procedures in regenerative medicine. This study is closely related to the psychophysical approach to human mental health therapy, in which we have proposed the use of dynamic patterns in electric and magnetic brain stimulation protocols, addressing electrochemical waves in neuro-astroglial networks.

## 1. Introduction

We have approached the concept of *Sentience*, in reference to universal dynamic patterns (amplitude-modulated waveforms; see [1,2]) that support conscious experience in living systems. These patterns are not intrinsically conscious, but their operations in neural tissues (e.g., forming synchronized rhythms in neural assemblies) endow living systems with the capacity of feeling and becoming conscious. We call *Sentiomics* the science responsible for the identification and preservation of these patterns, a task executed in scientific laboratories and natural environments, using the scientific method to explore mental potentialities intrinsic to natural processes.

In this paper, we establish conceptual foundations for an experimental research program aimed to preserve sentience, both in natural ecosystems and damaged brains. The main claim of the paper is about the possibility of providing an “education” to ‘in vitro’ brain organoids to allow the development of *sentience*, defined as the *capacity* of feeling [3,4,5,6]. In the beginning of the system’s self-organized growth, there is no cellular differentiation, and therefore, specialized sensory systems are absent, but each neuron (or proto-neuron) has a dendritic tree that can receive signals. We plan to develop biosensors, and to use already existing techniques from brain stimulation research (basically, electrical—with microelectrodes; and magnetic—with EM wave generators, using adequate frequencies and amplitudes, procedures), for the neural encoding of information during the early life of brain organoids.

The intended outcome of the research program is the ‘education’ of brain organoids grown in the Lab exposed to signals from nature, to induce the formation of adequate connections and dynamic processes necessary for the development of the capacity of feeling (sentience). According to [1,2], this capacity depends on the existence of an adequate substrate composed of electrochemical waves in neuro-astroglial networks and extracellular matrix.

A human brain organoid is a biological system composed of human neuronal and glial cells grown ‘in vitro’ (see, e.g., [7]), with affective, cognitive, and enactive potentialities, to be studied experimentally. Instead of the formative ontogenetic process of living systems in their niche, freely interacting with their ecosystems, brain organoids receive signals chosen by the experimenter during a training period. In this period, the experimenter should furnish the organoid with rich dynamic patterns, to activate the signaling networks existent in neural (neuronal and glial) tissues, making possible the formation of the necessary substrate for the expression of their mental potentialities.

In this paper, we present a preliminary approach to relevant issues likely to become the frontier of research and application in regenerative neuromedicine.

## 2. Epistemological and Bioethical Concerns

The generation of ‘in vitro’ brain organoids in the Lab raises relevant questions about their eventual affective, cognitive, and enactive capacities, as well as ethical concerns about human interactions in regenerative medicine and ‘post-humanist’ projects [8]. Before focusing on ethical issues [9], it is convenient to evaluate the biological viability of the emergence of sentience in brain organoids and possible usages in neuromedicine. Here, we argue that the emergence of sentience in brain organoids is not only possible, but also a probable consequence of the system’s neural structure (composed of networks of neurons and glial cells) *once an adequate type of ‘education’* (continued input of dynamic patterns) is given.

The concept of sentience and its implications should be clear from the start, to avoid premature worries about the bioethical status of ‘in vitro’ organoids. We do not assume that sentience is, or contains, a degree of consciousness, but instead we relate sentience with a *potentiality* for consciousness only. We define sentience as the *capacity* of: (a) perceiving stimuli, and (b) forming a feeling, which guides the response of the organism to stimuli. Therefore, in our conceptual framework, sentience is not the *conscious experience* of feeling (e.g., feeling pain), but refers to the biological substrate necessary for the experience. Only when the experience effectively happens (as in the example of feeling dizzy in a virtual roller coaster, discussed by Pereira Jr. [6]), engendering cognitive, affective and enactive functions, the corresponding bioethical concerns arise (e.g., is the organoid feeling pain?).

The study of conscious experience, and related bioethical issues, are not in the knowledge domain of *Sentiomics*. This science studies the *unconscious* dynamic patterns that form the biological substrate for the instantiation of feelings. It does not study the conscious experience of feeling. The distinction between *Sentiomics* and *Qualiomics*, discussed in the next section, has the goal of separating biological research on sentience from issues related to the study of qualitative conscious experiences, which are the subject of another knowledge area, called *Qualiomics*.

Making experiments with material substrates capable of feeling is equivalent to making experiments with neuron cultures, a trivial type of experiment carried out in many laboratories in the world, without special bioethical concerns related to animal or human experimentation. We agree with the claim that any neural network can generate sentience [10]. However, only when a developed neural system is “educated” and *consciously experimenting with feelings*, does the comparison with animals or plants, and the corresponding bioethical concerns, become relevant. In our research program with brain organoids, we are still not in this phase of the research. In the future, organoid consciousness may become a reality [11], and then, bioethical principles should become established for the social interaction of these systems with human society.

We also recall that bioethics is an enterprise of the scientific community as a whole, directed to establish universal principles. It is not a statement that each researcher or group of researchers make for each experiment with living systems. We prefer to wait until the rules for experimentation with mature brain organoids are established by the community, and then follow them in practice, instead of speculating in advance.

We claim that such an education of organoids to become sentient systems is necessary for the purposes of regenerative neuromedicine, e.g., growing neural tissues in vitro for transplantation to human brains in vivo. The reason why this is necessary is that the main function of neural tissues is to carry mental functions, which require a proper type of development for information processing in neuro-astroglial networks that compose the tissue. Only properly ‘educated’ brain organoids are likely to develop the electrochemical waves that (according to [1,2]) operate as a specialized substrate for the instantiation of feelings. In biology, structures are intimately connected to functions, and the functions of cells of the nervous system are cognitive, affective and enactive, all of them depending on sentience, the capacity of feeling [4]. We claim that the exposition of brain organoids to rich dynamic patterns (for instance, those registered from a natural environment such as the Amazon Rainforest) can elicit the type of electrochemical waves found in conscious systems, operating as a substrate for the instantiation of feelings.

## 3. Sentiomics and the Human Brain

Sentience has been approached as a psycho-biological phenomenon, corresponding to a cycle in living tissue composed of processes of chemical homeostasis in neural tissue (involving transmitters, modulators, hormones and peptides) that activate hydro-ionic waves (mostly calcium waves), and the feedback from these waves, controlling electrochemical homeostasis [3], The dynamic patterns of the waves in living tissue, both in animals and plants [1,2], make possible the conscious experience of feelings. Basic sensations, such as hunger and thirst, pleasure and pain, and mood states (depressed, euphoric) are (putatively) generated as temporal processes involving these electrochemical waves.

Ways of feeling are studied in two modalities:

(A) As the *universal set of patterns* of Sentience, which we call *Sentiomics*;
(B) As species-specific and individually different sets of *qualitative subjective experiences*, which we call *Qualiomics*.

*Qualiomics* is, of course, a difficult issue for conventional science, as stated in the “hard problem of consciousness” [12], because it leads to the much-discussed distinction of first- and third-person perspectives. The first-person perspective of individuals belonging to other species, or even to our species, is not accessible to scientific observation, measurement, and objective explanation.

There is an important difference between the capacity of feeling and emotion: while the first is a basic phenomenon possibly present in (almost) all living systems, the second is a higher-order phenomenon, corresponding to a dynamic cycle in which the affective drive that results from sentience is matched with affordances in the domain of the interaction of the agent with the environment [6], generating contextual *emotional feelings*, such as social emotions of love and hate, and individual emotions, such as being happy or sad. The study of conscious emotions belongs to the domain of *Qualiomics*, a field of knowledge constructed with first-person perspective approaches (as Introspective Psychology, Qualitative Research, and other forms of reporting subjective experiences).

*Sentiomics* is more amenable to a scientific (empirical, experimental, and hetero-phenomenological) treatment than *Qualiomics*. For instance, in our experimental work, we assume that dynamic patterns captured in real time by biosensors in the Amazon Rainforest relate to the sentience of the living beings that compose the ecosystem. We claim that the preservation of biological species is also the preservation of these universal forms of sentience, and vice-versa: the preservation of universal forms of sentience can also contribute to the preservation of the species.

*Sentiomics* becomes relevant in a historical moment when biological populations are under threat, modified in their ways of living, or extinct. We allude to the possibility of *preserving sentience* with the help of scientific measurements, artistic sensibility, and technological tools. This type of project can be interesting as a “safety net” for the preservation of data about the sentient ecosystems, and as an auxiliary tool for the mobilization of people’s environmental consciousness.

There are three types of conscious functions [13] carried out by the human brain: affective, cognitive, and enactive (concerned with the control of action). The study of mental health based on the neurosciences and psychology has focused mostly on cognitive and enactive functions. The study of affective processes is difficult because the neural substrates of feelings (from sensations to social emotions) are not local in the brain. Rather, they involve distributed and temporal processes related to the functions of glial cells (cells that, in the CNS, outnumber neurons, and that do not conduct nerve impulses).

‘Sentience’ refers to the unconscious neural processes that make us capable of feeling. As far as we know, only sentient beings are conscious. Being conscious is “feeling what happens” [14]. The neural bases of feelings are related to the control of homeostasis and allostasis by glial cells [3,4,5,6]. These cells were brought to scientific attention by means of the work of a group of scientists led by R. Douglas Fields. In his book “*The Other Brain*”, he stated: “The potential breakthroughs for medical science…are the most exciting frontier in glia research today. Diseases such as brain cancer and multiple sclerosis are caused by diseased glia. Glia plays an important role in such psychiatric illnesses as schizophrenia and depression and in neurodegenerative diseases such as Parkinson’s and Alzheimer’s. They are linked to infectious diseases such as HIV and prion disease (mad cow disease, for example) and to chronic pain. Scientists have discovered that glia can help repair the brain and spinal cord after injury and stroke” [15].

The generation of feelings in neural tissues involve temporal processes of homeostasis and allostasis [3] achieved by means of electrochemical slow waves of calcium ions inside astrocytes (the most common form of glial cell in the CNS), prompting currents of calcium and potassium ions into the extracellular milieu, where they modulate neural activity [16,17,18]. These slow waves and currents are not registered by conventional scalp EEG, or by technologies such as functional magnetic resonance imaging. They can, however, be imaged by invasive methods, such as optical imaging with two-photon microscopy or invasive electrodes during brain surgery.

Waking up involves a rise in the amplitude of these ionic waves and currents, together with other electrochemical processes. Nedergaard and colleagues have found evidence for what they call the ‘glympathic’ system, composed of channels for the circulation of cerebrospinal fluid in neural tissues. While we are awake and sentient, there is an increase in lactate levels in the glympathic pathways, whereas the lactate concentration decreases when we are in deep sleep [19].

The sensations, emotions, and affective states we experience depend on several factors. For instance, gastronomic experiences depend on our degree of hunger; experiencing the same food at different times and under different conditions may be variously pleasurable or disgusting, depending on the initial state of our digestive and neural systems. More generally, the quality of our feelings depends on the temporal dynamics of physiological processes involving mechanisms of homeostasis and allostasis [3].

The treatment of mental problems related to brain pathologies has been mostly through pharmacological interventions. Although these methods have improved in the last decades (see, e.g., the development of serotonin reuptake inhibitors), they are limited and, in some cases, produce adverse effects and/or lead to drug addiction. Most of them do not have the electrochemical medium as their target, but operate on neuron membrane receptors that are mostly involved with cognitive and enactive processes. One remarkable exception is the therapeutic use of lithium for treating bipolar disorder, because this exogenous ion can support the same functions as endogenous calcium ions in astrocytes.

The understanding of the dynamical patterns of sentience and their relationship with glial cells may allow the development of new pharmacological therapies (e.g., [20]) and also non-pharmacological therapies for the diseases of the brain and the mind [21,22]. Non-pharmacological techniques include, for instance, brain electro-magnetic stimulation to reduce abdominal pain [23] and electrical stimulation targeting astrocytes used against depression [24]. A possible therapeutic development of brain physical stimulation technology to treat affective disorders is direct brain (magnetic or ultrasound) stimulation with dynamic patterns, such as music or other types of bio-signals, considering that they involve the same type of temporal amplitude-modulated waveforms of the brain’s endogenous activity. It can be conjectured that we can directly stimulate the brain with dynamic patterns using devices that induce the amplitude-modulated signal (for instance, using a scalp-located device, such as the rotating magnets developed by Helekar et al. [25], or the input of chemical biosensors, as described in the next section). This would be an alternative to stimulation with non-informational sine waves (used in transcranial Direct or Alternating Current Stimulation, tDCS and tACS, respectively) or static EM fields (used in Transcranial Magnetic Stimulation, TMS). The patterns to be used in stimulation could be adjusted to the taste of the subjects, personalizing the information and making it possible to address specific types of affective states—instantiated in ionic waves and currents—instead of perturbing the whole neural tissue [26].

## 4. Building a Sentient and Creative Brain Organoid ‘In Vitro’

In the same path of therapeutic strategies targeting dynamic patterns of hydro-ionic waves embodied in neuro-astroglial networks, we propose to develop brain organoids for use in regenerative medicine, giving them a continued information-rich stimulus during an ‘educative’ period.

Receiving information about what happens is not sufficient for becoming conscious; besides receiving the information, the conscious system must also feel ‘what it is like’ to be informed. A machine that is not capable of feeling (such as a computer or a smartphone) only registers information without becoming conscious of this information. On the basis of the previous work we have carried out (and summarized above), we claim that to become conscious, a brain organoid should be continuously fed with rich dynamic patterns, to afford the development of neural (neuronal and glial) networks and patterns of activity compatible with sentience, the *capacity* of feeling, considered to be a condition for consciousness [4,5,6].

**Figure 1:**
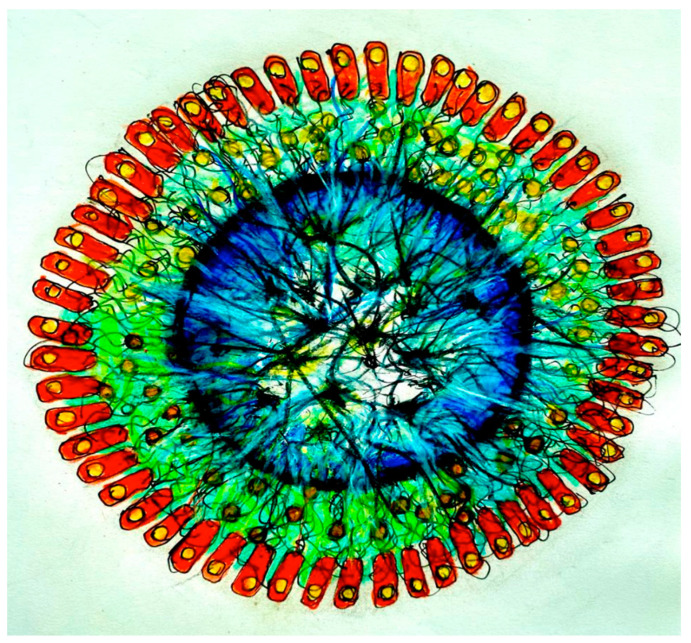
Coupling of Bacteria and Brain Organoid by Biosensors. A genetically engineered bacterial network (colored red) connected around a brain organoid (colored blue; the intensity corresponds to hypothetical neuron firings) by means of a bio-sensor composed of a synthetic interface device made of graphene (green) and golden quantum dots (yellow) to deliver natural bio-signals to the organoid. The bacteria have the role of transducing dynamic patterns from the Amazon Rainforest environment for the ‘education’ of the organoid.

In our experimental setting, bio-signals (molecular, chemical, bioelectric, and bio-electro-magnetic) from the Amazon Rainforest will be recorded on independent devices, according to the type of signal (e.g., molecular and chemical signals from microorganisms, sounds of birds, insects, amphibians, rain, wind, etc.). With the current tools of synthetic biology, we can build genetic circuits internalized in bacteria extracted from the Amazonian rhizosphere. Such biosensors will be able to transfer information from the patterns generated by Amazon plant and animal species and the physical environment to the organoids, mediated by an interface that uses graphene networks and ‘quantum dot’ technologies (Figure 1).

**Figure 2:**
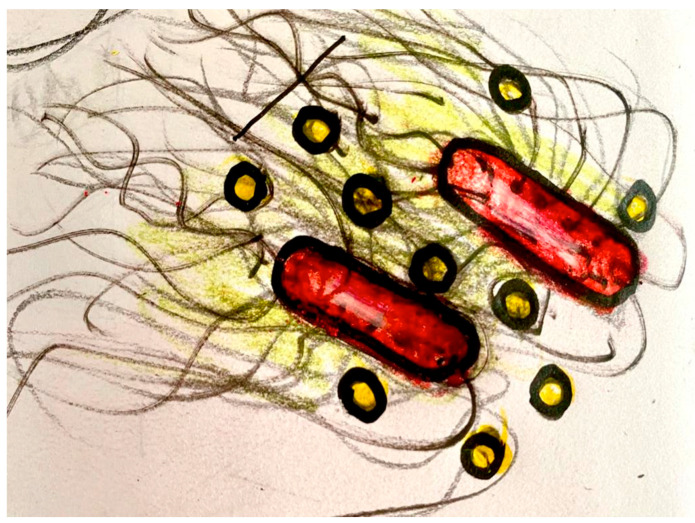
Synthetic device to connect Bacteria to Brain Organoids. The bacteria (colored red) grows curly fibers (black) that wrap (and are sustained by) the hexagonal structure of the graphene (not shown in the picture), forming a structure in which the golden quantum dots (yellow) flow, transmitting the information (dynamic patterns of the rainforest) to the brain organoid (see Figure 1).

Records will be compositionally treated and transmitted to the brain organoid via interfaces that generate or modulate Local Field Potentials. The interface with the brain organoid may be an array of implanted microelectrodes, a rotating and/or vibrating magnets, and/or biophysical or biochemical interfaces, delivering a temporally structured stimulation of bio-signals. In conventional settings, the signals, in a pulsed current, are supplied to the brain organoid with the proper voltage and amperage, shaping both the dendritic potentials of neurons and the calcium waves of astrocytes. In the synthetic interface, which may be used to deliver bio-signals from bacteria of the Amazon Rainforest to brain organoids, graphene and gold quantum dots are used for the communication of dynamic patterns (Figure 2). The bacterial network can be genetically engineered to capture specific types of bio-signals, using the CALTECH Biological Circuit Design (see http://be150.caltech.edu/2020/content/lessons/01_intro_to_circuit_design.html accessed on 29 November 2022) derived from the work of MIT researcher Chris Voigt and collaborators [27].

In the cultivated brain organoid, functional regions develop, based on genetic determinations and the types of input provided. The variety of bio-signals from the forest makes it possible to induce a number of specialized sensory regions, able to recognize specific types of dynamic patterns. These regions interact and generate an output signal in the effector region of the brain organoid (corresponding to the human motor system). In the input regions, the recorded signals are delivered, and in the output region, single-cell electrodes are inserted to record the respective spike trains.

In providing these signals for the brain organoid, we seek to induce the formation of circuits specialized in pattern recognition. For this, the forest signals will be organized according to, for instance—in analogy with musical patterns—rhythmic, “melodic”, “timbre”, and “harmonic” characteristics of electrochemical waves. Each of these compositional arrangements will be transmitted to a brain organoid region, to induce functional specialization during the training period. For example, considering the modality of audio recording, it is possible to organize the forest registers in four compositions: the first one, highlighting melodies, with birdsongs; another one, focusing on the rhythm, with sounds of raindrops; a third one, highlighting the variety of timbers produced by insects and amphibians; and a fourth one, highlighting the harmonic spectrum of the sound produced by the wind on tree leaves, and other sounds that present a rich spectrum of frequencies. This is only a simplified example, because a new generation of biosensors will be developed in the experiment to capture molecular, chemical, and microbiological dynamic patterns from the forest, using the bacterial synthetic biological device shown in Figure 1 and Figure 2.

In the training period, sensory specialization should be induced in the brain organoid. Considering Hebb’s Law, it is assumed that each region submitted to a type of stimulus will form interconnections specific to the information patterns present in the stimulus. In the process of Long-Term Potentiation, it is assumed that the connections formed will present a greater sensitivity to the type of pattern they were repeatedly exposed to. The patterns of connections formed at each specialized region putatively generate different types of dynamic patterns of electrochemical waves.

The training period corresponds to the time necessary for the consolidation of neural connections, through the plasticity of the system, which involves the formation of calcium currents and waves (respectively, in neurons and astrocytes), the activation of signal transduction pathways within neurons, gene regulation, the production of the growth factors of dendrites and axons, and the formation of new dendritic and axonal ramifications. The time required for the brain organoid to consolidate these memory traces should be evaluated during the experiment, as there are no available data that allow us to pre-determine the time of learning.

The generation of informational coherent responses to the input, and the development, of adaptive patterns of action (when the brain organoid is connected to effector parts, affording actions) may be rewarded with an increase in the supply of nutrients or modulators. In the output region, single cell electrodes can be used, capturing the axonal firing of neurons. During training, there is feedback from these spike trains to the sensory areas of the brain organoid. For this, we will make use of an artificial intelligence counting algorithm to evaluate the informational coherence of these spike trains, “rewarding” the system with nutrients (glucose) or transmitters that generate pleasurable sensations (for example, dopamine) when there is more consistency in the signals (consistency: maximized combination of variety and redundancy). The type of reward will be determined during the research.

After the training period, in which each region has established specialized connections forming neural circuits (in its interior and with the other regions), the experiment enters the second stage, the production of works by the brain organoid, through an interface that connects the output with a signal converter and a synthesizer. The spike trains generated by the brain organoid, spontaneously displaying—similar to any other axonal firing ‘in vivo’—the encoding of information patterns in frequency and phase, is connected through the signal converter with a synthesizer that generates virtual reality streams. In this part of the experiment, we assume that the various specialized sensory regions interact with each other, allowing the system to self-organize, generating a coherent output signal.

In the experimental setting, after the training period, the system will be exposed to new stimuli. Its responses can be recorded, using an interface with the human observer (e.g., a converter of the filtered binary signal—the spike trains generated by the brain organoid—to a synthesizer), generating a ‘virtual reality’ product for our evaluation—using psychological assessment protocols—of possible neural activity in the system. This is a task for *Qualiomics* research. Besides this ‘qualitative’ method, physiological biomarkers [4] can also be used to evaluate the mental activity of brain organoids.

Our working hypothesis for this phase of the research program is that the cognitive work produced by the brain organoid will convey a “feeling” that impacts the human receptor, which will then experience a ‘virtual reality’ exposition of the product. Considering that the brain organoid is generated from human cells, containing our species’ genome, there is a potential for brain organoids to develop in similar ways during the training period, allowing the emergence of creative responses to new stimuli. We predict the emergence of conscious experiences in the first-person perspective of the brain organoid, motivating the system to compose creative works, recombining the dynamic patterns previously presented. This prediction is based on the abovementioned fact that this type of system contains the same types of cells (neurons and astrocytes developed from stem cells) as the human nervous system. Experiencing a human-like feeling that motivates creativity, the brain organoid can generate cognitive responses with sentient qualities, to be evaluated by a human committee at the end of the experiment. At this phase of the research program, both the qualitative evaluation of the outputs of the system, and the bioethical concerns related to their use in human society, are issues that belong to the knowledge domain of *Qualiomics*.

## 5. Astrocytes in the Development of Brain Organoids and Regenerative Medicine

Astroglial cells and networks are the keys to theoretical and technological progress in the new fields of research Sentiomics and Regenerative Neuromedicine. As we have claimed above, and in several publications, astrocytes embody the hydro-ionic waves of *sentience*, the capacity of feeling, which is necessary for the emergence of consciousness. These waves also play a key role in the development of brain organoids (where they can develop [28]) and possible applications in Regenerative Neuromedicine.

The role of astrocytes in the development of neural tissues is well-known (see, for instance, https://www.biotechniques.com/cell-and-tissue-biology/using-astrocytes-to-speed-organoid-development/, accessed on 29 November 2022). Astrocytes participate in neuronal synapses, receiving signals from the neurons and modulating them back [16,29]. Neuronal synchrony induces the formation of astroglial hydro-ionic waves by means of constructive interferences [16]. These waves have been claimed to embody affective patterns of sentience [4] and participate in the brain’s large-scale integration of temporal patterns that provide the ‘signature’ of consciousness [30].

In brain organoids, the roles of astrocytes are the same as those in animal brains freely interacting with the environment, with the only difference being that the brain organoids are ‘in vitro’ entities (similar to Putnam’s famous story about “the brain in a vat”; see [31]). Once the system is provided with an artificial interface with the environment, as sketched in our previous sections, affording—with the help of the experimenter—“action-perception cycles” and an educational process similar to the experience of living systems interacting with their environment, it can develop sentience in a similar manner.

The results of this experimental program can have many applications in Regenerative Neuromedicine:

(1) In vitro studies of new drugs and physical (magnetic and electric stimulation) therapies used for ‘in vivo’ therapeutic procedures;
(2) Transplantation of neural tissue to damaged regions of the nervous system, highlighting the role of glial (‘glue’) cells: not only astrocytes, but also microglia [32];
(3) ‘In vitro’ studies of brain development (e.g., [33]) and regeneration (e.g., in cases of ischemia, hemorrhage, epileptic crises, traumatic lesions, cancer or aging degeneration diseases) and the testing of therapeutic resources [34,35,36,37].

## 6. Registering Signals of the Amazon Rainforest for Use in Brain Organoids’ ‘Education’

Exemplifying and advancing our reflections and practical actions towards the viability of *Sentiomics*, we report a scientific and artistic sub-project of recording data in the Amazon Rainforest, using it to preserve the “feeling of the forest”, and eventually induce them to other sentient beings, as human brain organoids, or to human sensibilization in artistic installations and museums of natural history.

In an age of glimpsing the possibilities of artificial intelligence, the rationality that led to the development of the machine and the industrial age also led to a disconnection of the human with his own senses and his environment. In this context, a scientific revival emerges, which seeks not a return to a primitive state, but a transcendence of the human-nature relationship *mediated* by the very intelligence of the machine. This opens the door for new multidimensional and multisensory explorations; for instance, projects to catch, process, and display life’s invisible dynamics. It aims to be a nature mediation system, approaching natural processes not apprehended by our senses, or facilitating the visualization of large datasets. More than just showing, it can correlate and predict phenomena.

For this purpose, we have captured a variety of signals of the forest in real time and compared these with previous registers. The data is “human knowledge” only in the sense that human beings place the sensors, build the computers that record them, detect the correlations, and help to generate an interface for public exposition of the results. According to the conceptual foundations of Sentiomics, the detected and analyzed dynamic patterns are universal, being present in all varieties of living systems, in different combinations and temporal sequences.

The project starts with data from the Amazon Rainforest ZF2 tower, Amazon Satellite Data, and the Massachusetts Living Observatory, in collaboration with the MIT Media Lab, comparing the dynamics of the 1500 y.o. forest with a recent regeneration area, analyzing the similarities and differences between conservation and restoration processes.

A system capable of displaying phenomena in real time is a new kind of interface for science. The scientific method seeks to store large datasets, and then further process and display them in the form of tables, diagrams and graphs. Our *Biobit Forest* system stores data from the past, learns from it, and can process new information. This makes it a communication interface with a natural phenomenon in real time, where events can be observed “in the act”.

Experiencing high complexity or high dimensionality of datasets represents a critical obstacle to reach new clusters of dynamical patterns in nature. Through a large amount of data already available from the last 40 years of the partner scientific institutions, we propose a computational processing project that finds complex correlations and patterns, creating an artificial intelligence capable of interpreting real-time phenomena. Past information, constantly updated, feeds the system. Computational intelligence processes data and looks for patterns, becoming capable of interpreting data in real time. The result is a scientific/artistic representation system that enables the multidimensional sensing of data. These environments have physical and sensory elements that reorganize into dynamic flows according to data input. Kinetic structures, geometric transformations, use of colors and sounds are some of the possibilities. The purpose is to create a language that decodes nature and expresses itself through sensory experiences. In sum, the *Biobit Forest* research affords a new paradigm in the scientist’s relationship to data, a new way of studying nature, by means of the elucidation of complex relations between crossed data, allowing the investigation of the role of the Amazon in global climate dynamics; and the comparison between data from a native forest and a regenerating area.

The preservation of lifeforms is usually addressed as a political and legal issue, focused on the continuity of biodiversity across human generations. Treves et al. [38] propose some ethical principles for biological preservation (“geocentrism, equitable consideration of non-humans, bio-proportionality, and intergenerational equity”) to guide the institution of courts “with constitutional powers to adjudicate the rights of futurity and non-humans against the rights of present humans who are threatening all life on Earth with our all-consuming use of natural resources” [38].

Besides the legal and political concerns, there is also an important philosophical issue at play within efforts to preserve lifeforms. The modification or extinction of a biological species is also—in a Monist perspective for which the material and mental aspects belong to the same reality—the modification or extinction of a form of sentience and its related dimensions. For instance, we are interested in the preservation of biological species, such as the Panda, not only because of their biological (genetic, physiological, ecological) richness, and our (human) appreciation of them, but also because they are sentient beings, with ways of feeling that we can inter-subjectively share with them.

Each biological species, besides having proper genes, proteins, metabolic processes, and action schemes to be preserved, also has sentience. Does “just preservation” of the respective populations assure the preservation of their ways of feeling? [39] notes that preservation involves a holistic view of nature. Considering changes caused by the human destruction of ecosystems, “just preservation” of biological beings does not imply the preservation of sentience, because the ways of feeling are closely related to ecological interactions; changing the ecosystem or moving the biological individual to a completely different environment, the dynamic patterns of the sentience of biological populations and individuals are likely to change.

In our foresight, *Sentiomics* has the potential to be the next step in a sequence of developments for the study and promotion of biodiversity and natural forms of mentality, moving forward in relation to successful projects of identification and preservation of genomes and proteomes. Although the artificial intelligence of a machine is not sentient, it can be used as a *medium* to organize data that *preserves the correlations found in sentient systems*. The structured database can be used in other, different biological substrates, such as the brain organoid in Regenerative Neuromedicine, to induce sentience, in an interdisciplinary effort that integrates ecosystemic information richness and human neuromedicine, to promote the health of both—the ecosystem and the damaged human brain.

## 7. Concluding Remarks

The reviewed scientific information and theoretical proposals afford an experimental program to investigate the neural potentialities of brain organoids trained with signals from the Amazon Rainforest ecosystem, with the following goals:

(1) Record and preserve bio-signals from the Amazon Rainforest;
(2) Use the informational variety of these dynamic patterns to generate a repertoire of learning in brain organoids;
(3) Induce, in brain organoids, the emergence of sentience, evaluated by means of biomarkers, according to the proposal formulated by [4];
(4) Generate works from the responses of the brain organoids, to be evaluated by human persons, and eventually, they can also be part of exhibitions related to the environmental issue;
(5) Enable the use of the brain organoid in “post-humanist” projects for the preservation of sentience, both demonstrating the mentality intrinsic to nature and contributing to human “reconnection” with the information richness of nature;
(6) Generation of brain organoids for the treatment of lesions, neurological illnesses, and degenerative diseases affecting the nervous system.

Beyond these achievements, we also predict that the transfer of dynamic patterns from biosensors to brain organoids will make them capable of learning and acquiring habits. In the next phase of the investigation, organoids coupled with biosensors placed in the Amazon Rainforest, instead of the Lab, will be able to behave as a hybrid intelligence; that is, they will be able to acquire specific functions to detect and feel still unknown types of biochemical and biophysical information.
